# Non-climacteric fruit development and ripening regulation: ‘the phytohormones show’

**DOI:** 10.1093/jxb/erad271

**Published:** 2023-07-14

**Authors:** María Florencia Perotti, David Posé, Carmen Martín-Pizarro

**Affiliations:** Departamento de Mejora Genética y Biotecnología, Instituto de Hortofruticultura Subtropical y Mediterránea ‘La Mayora’ (IHSM), Universidad de Málaga - Consejo Superior de Investigaciones Científicas, Departamento de Biología Molecular y Bioquímica, Facultad de Ciencias, UMA, Málaga, Spain; Departamento de Mejora Genética y Biotecnología, Instituto de Hortofruticultura Subtropical y Mediterránea ‘La Mayora’ (IHSM), Universidad de Málaga - Consejo Superior de Investigaciones Científicas, Departamento de Biología Molecular y Bioquímica, Facultad de Ciencias, UMA, Málaga, Spain; Departamento de Mejora Genética y Biotecnología, Instituto de Hortofruticultura Subtropical y Mediterránea ‘La Mayora’ (IHSM), Universidad de Málaga - Consejo Superior de Investigaciones Científicas, Departamento de Biología Molecular y Bioquímica, Facultad de Ciencias, UMA, Málaga, Spain; INRAE, France

**Keywords:** ABA, grapevine, non-climacteric, phytohormone, ripening, strawberry

## Abstract

Fruit ripening involves numerous physiological, structural, and metabolic changes that result in the formation of edible fruits. This process is controlled at different molecular levels, with essential roles for phytohormones, transcription factors, and epigenetic modifications. Fleshy fruits are classified as either climacteric or non-climacteric species. Climacteric fruits are characterized by a burst in respiration and ethylene production at the onset of ripening, while regulation of non-climacteric fruit ripening has been commonly attributed to abscisic acid (ABA). However, there is controversy as to whether mechanisms regulating fruit ripening are shared between non-climacteric species, and to what extent other hormones contribute alongside ABA. In this review, we summarize classic and recent studies on the accumulation profile and role of ABA and other important hormones in the regulation of non-climacteric fruit development and ripening, as well as their crosstalk, paying special attention to the two main non-climacteric plant models, strawberry and grape. We highlight both the common and different roles of these regulators in these two crops, and discuss the importance of the transcriptional and environmental regulation of fruit ripening, as well as the need to optimize genetic transformation methodologies to facilitate gene functional analyses.

## Teaser

We review recent advances in our understanding of the molecular regulation of non-climacteric fruit ripening at the hormonal level, with a special focus on strawberry and grapevine as model species.

## Introduction

Fleshy fruit ripening is a complex and tightly regulated process that involves biochemical and physiological changes including: (i) alterations in color due to the disassembly of the photosynthetic system and the replacement of chlorophylls with anthocyanins and/or carotenoids; (ii) changes in texture as a result of cell wall degradation and loss of cell turgor; and (iii) an alteration of the aroma and flavor due to the accumulation of sugars, organic acids, and volatile compounds. These changes make the fruit more appealing for consumption, allowing dispersal of seeds once they are mature. Fleshy fruit species are traditionally classified as climacteric and non-climacteric based on the hormonal mechanisms that control the ripening process. Climacteric fruits, such as tomatoes, apples, or bananas, show a burst of respiration and a rapid increase in ethylene production at the onset of ripening, which acts as a key signal for the initiation and coordination of the process. On the other hand, in non-climacteric fruits, such as strawberry, grape, sweet cherry, and lychee, among others, ethylene seems to play a minor role and does not necessarily initiate the fruit ripening. Instead, abscisic acid (ABA) is considered the key hormone regulating this process (Chai *et al.,* 2011; Jia *et al.,* 2011). However, other hormones, including brassinosteroids (BRs) and methyl jasmonate, have been shown to play important roles during ripening regulation in non-climacteric fruits (Chervin *et al.,* 2004; Symons *et al.,* 2006; Chai *et al.,* 2013; Villarreal *et al.,* 2016; García-Pastor *et al.,* 2019; Zuñiga *et al.,* 2020). Although it will not be the focus of this review, there are various other important players and processes involved in the regulation of the non-climacteric ripening process besides hormones, including transcription factors (TFs) (Sánchez-Gómez *et al.,* 2022), sucrose signaling (Jia *et al.,* 2013a), DNA methylation (Shangguan *et al.,* 2020; Martínez-Rivas *et al.,* 2022), and changes in osmotic potential (Jia *et al.,* 2020), among others, as well as external stimuli such as light and temperature (Abeysinghe *et al.,* 2019; Twitchen *et al.,* 2021).

Two plant species have been used as the main experimental systems in the study of non-climacteric fruit ripening, namely strawberry [both the cultivated species *Fragaria* × *ananassa* (octoploid) and the woodland strawberry *F. vesca* (diploid)] and grape (*Vitis vinifera*), which are totally different in their fruit structure. Grapes develop ovary-derived berries, which are composed of seeds and three major tissues: skin or exocarp, pulp/flesh or mesocarp, and endocarp (Hardie *et al.,* 1996). In contrast, strawberry fruit is an aggregate fruit (achenetum), where the fleshy part is originated from the enlargement of the floral receptacle, which is connected to the actual fruits, called achenes, located on its surface (Liu *et al.,* 2020). For convenience, however, strawberries will be called ‘fruits’ throughout the text.

These two fruit crops are of great economic importance worldwide. Deciphering the molecular and environmental regulation of their fruit ripening is therefore a high priority to understand the biochemical changes that take place during this process and how they are regulated, and facilitate the control and improvement of fruit-related quality traits. Although there is no available mutant collection for either of these crops, gene functional analysis can be performed by either transient or stable transformation, although it is faster and easier in strawberry than in grapevine. The majority of studies in grape involve the application of different treatments or hormones at different stages of fruit development, followed by exhaustive sampling and subsequent transcriptomic analyses to investigate their effect on ripening (Zillioto *et al.,* 2012; Chai *et al.,* 2014; Cheng *et al.,* 2015; Pilati *et al.,* 2017), an approach that has also been commonly followed in strawberry (summarized in Table 1). Furthermore, transcriptomic and metabolomic analyses throughout strawberry and grape fruit development and ripening have been essential to decipher the molecular changes that occur during these processes, allowing the identification of related changes in gene expression or levels of metabolites (Kang *et al.,* 2013; Härtl *et al.,* 2017; Massonnet *et al.,* 2017; Sánchez-Sevilla *et al.,* 2017; Wang *et al.,* 2017; Fasoli *et al.,* 2018; Gu *et al.,* 2019). Here we review classic and more recent studies that have shed light on the hormonal regulation of non-climacteric fruit, focusing mainly on these two model species.

**Table 1.** Treatments with hormonal and inhibitor compounds in strawberry and grape fruits.

Strawberry/grape ripening					
Species	Hormones	Concentration	Timing of application	Effect on ripening	Species and references
	**AUXINS**				
**Strawberries**	**Synthetic auxins**	1 mM IAA	At green stage	Delay	*F. vesca* ‘Ruegen’. Gu *et al.* (2019)
		500 µM NAA	At green stage	Fruit growth	*F. vesca* ‘Yellow Wonder’. Liao *et al.* (2018)
		50 µM NAA	Emasculated flowers	Fruit growth	*F. vesca* ‘Yellow Wonder’. Kang *et al.* (2013)
		1 mM NAA	Green deachened fruits	Inhibit ripening	*F.* × *ananassa* cv. ‘Brighton’. Given *et al.* (1988)
		1 mM 2,4-D	Green deachened fruits	Inhibit ripening	*F.* × *ananassa* cv. ‘Brighton’. Given *et al.* (1988)
		100 ppm BNOA	Green deachened fruits	Fruit growth	*F.* × *ananassa* cv. ‘Marshall’. Nitsch (1950)
**Grapes**	**Synthetic auxins**	20 ppm BTOA	6–8 weeks after flowering	Delay 2 weeks	*V. vinifera* cv. ‘Shiraz’. Davies *et al.* (1997)
		50 mg l^–1^ NAA	Before véraison	Delay 15 d	*V. vinifera* cv. ‘Riesling’. Böttcher *et al.* (2012)
		200 mg l^–1^ NAA	Before véraison	Delay 10 d	*V. vinifera* cv. ‘Merlot’, Zillioto *et al.,* 2012
		50 mg l^–1^ NAA	Before véraison	Delay 10 d	*V. vinifera* cv. ‘Shiraz’. Böttcher *et al.* (2011)
		200 mg l^–1^ NAA	At ripening	Inhibit ripening	*V. vinifera* cv. ‘Cabernet Sauvignon’. Jeong *et al.* (2004)
	**GIBBERELLINS**				
**Strawberries**	**GA**_**3**_	500 μM	1 day after pollination (DAP)	Promote fruit elongation	*F. vesca* ‘Yellow Wonder’. Liao *et al.* (2018)
	**Inhibitor PAC**	100 μM	1 DAP	Inhibit fruit elongation	*F. vesca* ‘Yellow Wonder’. Liao *et al.* (2018)
**Grapes**	**GA**_**3**_	3/5/7 mg l^–1^	20 d and 15 d pre-flowering	Promote color and anthocyanins	*V. vinifera* cv. ‘Syrah’. Xie *et al.* (2022)
		100 mg l^–1^	12 d pre-flowering	Promote berry color	*Vitis labrusca*×*V. vinifera* cv. ‘Kyoho’. Cheng *et al.* (2015)
		30 mg l^–1^	12 d after flowering	Promote berry enlargement	*V. vinifera* cv. ‘Centennial Seed-less’. Chai *et al.* (2014)
		500 mg l^–1^	Weekly	Increase total grapevine mass	*V. vinifera* cv. ‘Malbec’. Moreno *et al.* (2011)
	**Inhibitor PAC**	200 mg l^–1^	Weekly	Reduce total grapevine mass	*V. vinifera* cv. ‘Malbec’. Moreno *et al.* (2011)
	**BRASSINOSTEROIDS**				
**Strawberries**	**EBR**	400 mM	At green stage	Promote fruit color	*F.* × *ananassa* cv. ‘Akihime’. Chai *et al.* (2013)
	**Inhibitor BZ**	200 mM	At green stage	Delay fruit color	*F.* × *ananassa* cv. ‘Akihime’. Chai *et al.* (2013)
**Grapes**	**EBR**	0.40 mg l^–1^	One time during véraison	Promote	*V. vinifera* cv. ‘Yan73’ and ‘Cabernet Sauvignon’. Xi *et al.* (2013); Xu *et al.* (2015)
		40 ng µl^–1^	Four time points	Promote	*V. vinifera* cv. ‘Cabernet Sauvignon’. Symons *et al.* (2006)
	**Inhibitor BZ**	1.31 mg l^–1^	One time during véraison	Delay	*V. vinifera* grape cv. ‘Cabernet Sauvignon’. Xu *et al.* (2015)
		2 µg µl^–1^	Four time points	Delay	*V. vinifera* cv. ‘Cabenet Sauvignon’. Symons *et al.,* 2006
	**Ethylene**				
**Strawberries**	**Ethephon**	13 mM	At green stage	Delay anthocyanins	*F. chiloensis*. Figueroa *et al.* (2021)
		2 mM	At white stage	Changes in cell wall	*F.* × *anassa* cv. ‘Toyonoka’. Villarreal *et al.* (2016)
	**Inhibitor 1-MCP**	1 µl l^–1^	At white stage	Changes in cell wall	*F.* × *ananassa* cv. ‘Toyonoka’. Villarreal *et al.* (2016)
**Grapes**	**Ethephon**	144 mg l^–1^	Once or twice pre-véraison time	Delay	*V. vinifera* cv. ‘Shiraz’. Böttcher *et al*. (2013b)
		500 µM	2 weeks before véraison	Promote	*V. vinifera* cv. ‘Muscat Hamburg’. Sun *et al.* (2010)
		4 µl l^–1^	1 h or 24 h, 8 weeks after flowering	Increase berry diameter	*V. vinifera* cv. ‘Cabernet Sauvignon’. Chervin *et al*. (2008)
	**Inhibitor 1-MCP**	5 µl l^–1^	1 week before véraison	Delay	*V. vinifera* cv. ‘Muscat Hamburg’. Sun *et al.* (2010)
		4 µl l^–1^	Various times following full bloom	Delay	*V. vinifera* cv. ‘Cabernet Sauvignon’, Chervin *et al*. (2004)
	**Inhibitor AVG**	125 mg l^–1^	Twice pre-véraison time	Promote	*V. vinifera* cv. ‘Cabernet Sauvignon’, Böttcher *et al*. (2013a)
	**ABSCISIC ACID**				
**Strawberries**	**ABA**	1 µM	At green stage	Promote	*F.* × *ananassa* cv. ‘Toyonoka’. Li *et al*. (2019)
		95 µM	At green stage	Promote	*F.* × *ananassa* cv. ‘Benihoppe’. Luo *et al*. (2019)
		100 µM	1 DAP	Inhibit fruit growth	*F. vesca* ‘Yellow Wonder’. Liao *et al*. (2018)
		0,5 µM	At green stage	Promote	*F.* × *ananassa* cv. ‘Fugilia’. Jia *et al.* (2011)
		50 mM DMSO	At green stage	Promote	*F.* × *ananassa* cv. ‘Fugilia’. Jia *et al.* (2011)
	**Inhibitor compounds**	100 µM NDGA	At green stage	Delay	*F.* × *ananassa* cv. ‘Toyonoka’. Li *et al.* (2019)
		100 µM NDGA	At green stage	Delay	*F. vesca* ‘Ruegen’. Gu *et al.* (2019)
		50 µM fluridone	At green stage	Inhibit	*F.* × *ananassa* cv. ‘Fugilia’. Jia *et al.* (2011)
**Grapes**	**ABA**	0.2 mM	Hard green	Promote	*V. vinifera* cv. ‘Pinot Noir’. Pilatti *et al.* (2017)
		50/100 mg l^–1^	Hard green	Promote	*V. vinifera* cv. ‘Carménère’. Villalobos-González *et al*. (2016)
		500 mg l^–1^	At ripening	Promote	*V.vinifera*×*V. amurensis* ‘Beihong’. Wang *et al.* (2016)
		300 ppm	At ripening	Promote	*V. rotundifolia* Alachua and Noble. Sandhu *et al.* (2011)
		400 mg l^–1^	At ripening	Promote	*V. vinifera* cv. ‘Cabernet Sauvignon’. Koyama *et al*. (2010)
		0.76 mM	At ripening	Promote	*V. vinifera* cv. ‘Cabernet Sauvignon’. Giribaldi *et al*. (2010)
		250/500/1000 mg l^–1^	At ripening	Promote	*V. vinifera* cv. ‘Redglobe’ grapevines. Peppi *et al*. (2007)
		150/300 µl µl^–1^	At ripening	Promote	*V. vinifera* cv. ‘Crimson Seedless’ grapevines. Cantín *et al.* (2007)
		1000/2000 mg l^–1^	At ripening	Promote	*V. vinifera* cv. ‘Flame Seedless’ grapevines. Peppi *et al*. (2006)
		1000 mg l^–1^	At ripening	Promote	*V. vinifera* cv. ‘Cabernet Sauvignon’. Jeong *et al.* (2004)
		1000 ppm	At ripening	Promote	*V. vinifera* cv. ‘Kyoho’ grape plants. Ban *et al.* (2003)

AUX, auxins; IAA, indole-3-acetic acid; NAA, naphthalene-1-acetic acid; 2,4-D, 2,4-dichlorophenoxyacetic acid; BNOA, beta-naphthoxyacetic acid; GA/GA_3_, gibberellic acid; PAC, paclobutrazol; BR, brassinosteroid; EBR, 24-epibrassinolide; BZ, brassinazole; 1-MCP, 1-methylcyclopropene; AVG, aminoethoxyvinylglycine; ABA, abscisic acid; NDGA, nordihydroguaiaretic acid.

## Fruit development and ripening: stages and consequences. ‘The set list’

**Fig. 1.**
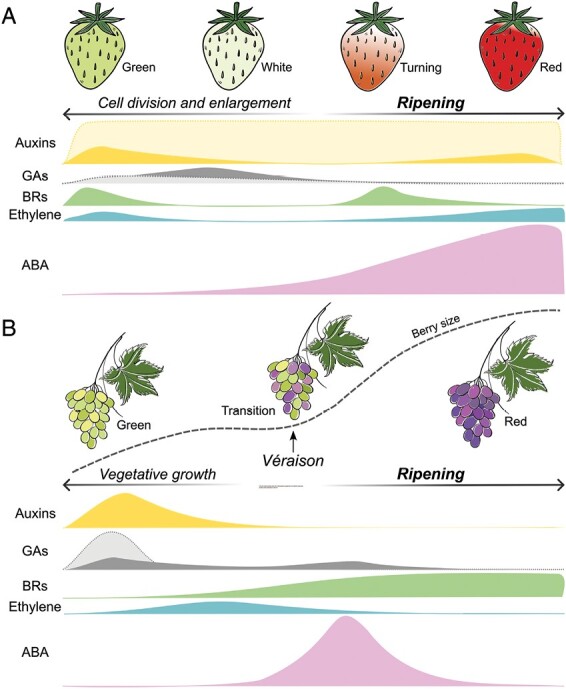
Schematic model of strawberry (A) and grape berry (B) developmental and ripening stages and the changes in hormone content from green to ripe fruit. Dotted areas represent hormone levels at strawberry achenes (A) and grape berry seeds (B). Filled areas represent receptacle/pulp or entire fruits.

Strawberry and grape development and ripening are classified into stages that differ between these two species. In the case of strawberry, there are four main stages defined by the color of the receptacle: green (which can also be subdivided into small, middle, and large green stages), white, turning, and red. Cell division and expansion occur in the first two stages, while the latter two are the actual ripening stages (Fig. 1A) (Symons *et al.,* 2012). In the case of grape, fruit development and ripening can be separated into three stages: two sigmoidal phases separated by a lag phase (Fig. 1B). The first stage (phase I) comprises fruit set until the formation of green hard berries. During this time, rapid cellular division and enlargement take place. Then, a lag phase (phase II) is distinguished by a pause in berry growth. Then the last stage (phase III) starts with the so-called véraison, corresponding to the onset of ripening (Conde *et al.,* 2007).

## Auxins and gibberellins: ‘the backstage crew’

Auxins are known to induce fruit growth in many different plant species, including climacteric fruits, such as tomato and apple (Serrani *et al.,* 2008; Devoghalaere *et al.,* 2012), and non-climacteric fruits, including both strawberries (Nitsch, 1950; Kang *et al.,* 2013; Liao *et al.,* 2018) and grapes (Godoy *et al.,* 2021). In strawberries, auxins are synthesized primarily within the achenes and play a central role in the early stages of development. This was supported by classic experiments in *F.* × *ananassa* where removal of the achenes impaired receptacle development, which was restored upon exogenous application of the synthetic auxin, naphthoxy-3-acetic acid (NOA) (Nitsch, 1950). Furthermore, treatment of *F. vesca* open flowers with another synthetic auxin, naphthalene-1-acetic acid (NAA), promoted fruit growth in both width and length (Liao *et al.,* 2018). An opposite role of auxin at later stages is also well established, since it inhibits non-climacteric ripening (Given *et al.,* 1988; Davies *et al.,* 1997). More recently, deachenization in fully developed strawberry fruits was found to promote ripening of the receptacle, but not when lanolin paste with indole-3-acetic acid (IAA), the most abundant natural auxin in strawberry, was applied, supporting the inhibitory role of auxin in this process (Gu *et al.,* 2019).

Different patterns of IAA accumulation have been observed in strawberry fruits. A maximum in the level of IAA was found at the early green stages of entire *F*. × *ananassa* and *F. vesca* fruits, which declined as fruit growth and ripening progressed (Symons *et al.,* 2012; Liao *et al.,* 2018; Upadhyay *et al.,* 2023). IAA patterns differed when achenes and receptacles of *F. vesca* fruits were analyzed separately. Using this approach, Gu *et al*. (2019) found much higher IAA levels in achenes than in receptacles, and that they remained constant throughout achene development and ripening. In contrast, IAA levels in receptacles were very low (Fig. 1A).

The most abundant natural auxin in grapes is also IAA, with an important role in enhancing a high rate of cell division and enlargement in the early phases of grape development. IAA has been detected in both flesh and seeds, increasing during the initial times of the vegetative phase, but gradually declining to very low levels during the middle of stage II, with a relatively low concentration at véraison (Fig. 1B) (Gouthu and Deluc, 2015). The reduced IAA levels are maintained in further stages due to an auxin inactivation mechanism, in which *GH3* genes (*GH3-1* and *GH3-2*), coding for IAA-amido synthetases, which conjugate IAA to amino acids, increase their expression in both the skin and flesh of grape fruits at the beginning of véraison (Böttcher *et al.,* 2011; Ziliotto *et al.,* 2012; Dal Santo *et al.,* 2020). The detection of IAA–amino conjugates together with low levels of free IAA at the onset of maturation, and immediately afterwards, has also been described in strawberry (Archbold and Dennis, 1984; Upadhyay *et al.,* 2023), and in other climacteric fruits, such as tomato (Böttcher *et al.,* 2010), banana (Purgatto *et al.,* 2002), and muskmelon (Dunlap *et al.,* 1996), suggesting that this is a conserved ripening-related process in non-climacteric and climacteric crops.

The inhibitory role of auxins in ripening has also been widely demonstrated in grapevine. For example, treatment of grape berries cv. ‘Shiraz’ with the synthetic auxin-like compound benzothiazole-2-oxyacetic acid (BTOA) delayed the beginning of ripening by 2 weeks and decreased anthocyanin accumulation due to the down-regulation of structural genes of the pathway, such as *chalcone synthase* (*CHS*) and *UDP-glucose:flavonoid 3-O-glucosyl transferase* (*UFGT*), as well as a reduction in sugar accumulation, berry softening, and ABA levels (Davies *et al.,* 1997). This delay has also been observed in other grapevine cultivars treated with NAA, such as ‘Shiraz’, ‘Riesling’, and ‘Merlot’, (Böttcher *et al.,* 2011, 2012; Ziliotto *et al.,* 2012). Interestingly, this alteration of the progress of ripening can be exploited to modify the quality of grape berries. Thus, significant differences were detected in the properties and volatile compounds of wine made from control and NAA-treated fruit, indicating that this kind of treatment might be used to manipulate grape and wine composition (Böttcher *et al.,* 2012).

Like auxins, gibberellic acid (GA) promotes fruit growth in both climacteric and non-climacteric fruits (Serrani *et al.,* 2008; Kang *et al.,* 2013; Chai *et al.,* 2014; Liao *et al.,* 2018). In strawberry, however, GA has a different role from that of auxins during the first stages of development. Treatments with GA_3_ of *F. vesca* fruits mainly promoted fruit elongation, but not widening as in the case of auxin, suggesting a more general role of the latter in receptacle development than that of GAs (Liao *et al.,* 2018). This is also supported by the results of treatments in which the GA biosynthesis inhibitor paclobutrazol (PAC) was included, showing that auxin promotes fruit growth in both a GA-dependent and -independent manner (Liao *et al.,* 2018). GA follows a trend of accumulation in entire *F.* × *ananassa* fruits that is similar to auxins, but is displaced slightly later in time, peaking at the large green stage (Fig. 1A). This is consistent with the role of auxin in promoting GA biosynthesis, not only in strawberries but also in other species (Serrani *et al.,* 2008; Dorcey *et al.,* 2009). Supporting the role of auxin promoting GA, fruits treated with NAA showed an increased expression of genes involved in GA synthesis, including several *GA20ox* and *GA3ox* genes, as well as a suppression of those with a negative role in GA signaling, such as *GAI*, *RGA1*, and *RGL3* (Liao *et al.,* 2018).

The connection between achenes and receptacles has been shown to be of great importance for fruit development based on transcriptome data. Thus, genes involved in auxin biosynthesis such as *YUC5*, *YUC11*, and *TAR1*, as well as GA biosynthesis genes, including *GA20ox3*, and *GA3ox3*, *4*, *5*, and *6* have been found to be mainly expressed in achenes but not in receptacles (Kang *et al.,* 2013). However, the signaling components for auxin and GA were more highly and specifically expressed in receptacles, which supports a role for the achenes as the source of these phytohormones, and the receptacle as the responding tissue (Kang *et al.,* 2013).

GAs also have an important role in the early phases of grape berry development, enhancing the rate of cell division and enlargement. Seeds are the main source of GAs (Pérez *et al.,* 2000), so GA is commonly applied to increase berry size and cluster length, especially in seedless table grape and raisin production (Acheampong *et al.,* 2017). This enlargement of the berries following GA treatment may be due to GA-dependent changes in the expression of genes involved in cell wall modification and the cytoskeleton (Chai *et al.,* 2014). GAs have a similar accumulation trend to auxin during grape development, increasing at the initial developmental stages and declining throughout development and ripening (Conde *et al.,* 2007; Wang *et al.,* 2020). Consistent with this accumulation pattern, a transcriptome analysis during grape development showed that the GA biosynthetic genes *VvGA20ox1-1*, *VvGA3ox4-1*, and *VvGA2ox1-1* and the GA receptor gene *VvGID1B* were highly expressed at the green stage, while declining in subsequent stages (Wang *et al.,* 2020). Interestingly, the expression of genes coding for the GA repressor DELLA proteins also peaked at this stage. Since the GA biosynthetic genes are targets of DELLA proteins (Zentella *et al.,* 2007), this co-expression pattern suggests a negative feedback to regulate the GA content during berry development (Wang *et al.,* 2020). However, and in contrast to strawberries, GA levels show a slight increase at mid stages of ripening (Fig. 1B) (Pérez *et al.,* 2000). Interestingly, GA_3_ treatment of grapevines before flowering promotes an earlier berry coloration, as well as an increase in reducing sugars, total phenolic, tannin, and total anthocyanin contents (Moreno *et al.,* 2011; Cheng *et al.,* 2015; Xie *et al.,* 2022). Together, these data may support a putative role for GAs in the last steps of the ripening process. Whether this accelerated ripening upon GA treatment is the consequence of a faster berry development or a direct effect on ripening-related processes is not clear. Therefore, further studies are needed to understand the role of the increase in GA levels at the late stages of ripening.

Of special interest is the *fleshless berry* (*flb*) mutation. This mutation impairs the pericarp development of the grape fruit, reducing its size at ripening due to an impaired division and differentiation of cells in the inner mesocarp, without any effect on fertility and seed size and number. Strikingly, treatments with GA, the synthetic cytokinin 6-benzyl-aminopurine (6-BAP), and IAA failed to reverse this phenotype, suggesting an impaired hormone signal reception or transduction in this mutant (Fernandez *et al.,* 2006). However, genes encoding TFs, namely *ATHB13*, *PISTILATTA*, and *YABBY2*, were differentially expressed, suggesting that they perform an important role in early grape fruit development (Fernandez *et al.,* 2007).

## Brassinosteroids: ‘a support artist’ with differing roles in strawberry and grapevine ripening

BRs are steroidal plant hormones that are essential for normal plant growth, related to cell division and elongation, vascular differentiation, reproductive development, and pathogen and abiotic tolerance (Nolan *et al.,* 2020). It is known that exogenous BRs stimulate ripening in climacteric fruits such as tomato (Vardhini and Rao, 2002), mango (Zaharah *et al.,* 2012), and persimmon (He *et al.,* 2018), increasing the ethylene levels. The same positive effect has been described in grapes. Thus, the exogenous application of the most bioactive BR, 24-epibrassinolide (EBR) or the BR biosynthesis inhibitor brassinazole (BZ) before véraison significantly stimulated or delayed ripening by increasing or decreasing the concentration of total soluble solids (TSS), respectively (Symons *et al.,* 2006). EBR treatment has been shown to promote grape berry ripening, modifying the expression of sugar transporters and invertases, as well as the activity of sugar metabolic enzymes, enhancing their TSS and reducing the sugar content (Xu *et al.,* 2015). This treatment also reduced the titratable acid content in the juices and increased the average berry weight (Xi *et al.,* 2013; Xu *et al.,* 2015). Enhanced activities of enzymes involved in the biosynthesis of anthocyanins, such as phenylalanine ammonia-lyase (PAL) and UFGT, have also been reported upon exogenous EBR application, resulting in an increased accumulation of the total anthocyanins in the berry skin (Xi *et al.,* 2013). Consistent with these effects of exogenous BR treatments, a rise in the levels of the endogenous bioactive BR castasterone (CS) and its direct precursor 6-deoxocastasterone has been detected at the onset of ripening, which coincides with a higher expression of BR biosynthesis (*VvDWFl*) and receptor (*VvBRI1*) genes (Symons *et al.,* 2006). All these data support an important positive contribution of BRs at the onset of grape fruit ripening.

Changes in BR content during *F*. × *ananassa* fruit development have also been described; however, the function of this hormone in strawberry is still poorly explored. CS levels showed a peak in flowers followed by a dramatic drop during fruit development and ripening (Fig. 1A), suggesting a possible role during the early developmental stages but not during the ripening process (Symons *et al.,* 2012). A similar pattern in which BRs are more abundant at earlier stages in deachened receptacles was found shortly afterwards by Chai *et al.* (2013). Nevertheless, this work showed that, as found in grapevine, EBR and BZ application to strawberries accelerated and delayed the fruit coloration, respectively. Furthermore, the down-regulation of the BR receptor gene *FaBRI1* by virus-induced gene silencing (VIGS) resulted in a lack of fruit coloration, suggesting a positive role for BRs in promoting strawberry fruit ripening (Chai *et al.,* 2013). Therefore, further studies need to be performed to better understand the role of BRs in strawberry fruit development and ripening.

## Ethylene: ‘the surprise act’

While ethylene is the major hormone controlling fruit ripening in climacteric fruits, no such central role has been described in non-climacteric fruits, although several studies suggest that it also plays a positive role in their ripening. In the octoploid strawberry fruits, ethylene content increases at the green stage, followed by a reduction at the white stage and increasing again at the last stages of ripening, which, interestingly, is accompanied by an enhanced respiration rate, as occurs in climacteric fruits at the onset of ripening (Fig. 1A) (Iannetta *et al.,* 2006). A similar increase from the turning to the ripe stage was also found for the ethylene precursor 1-aminocyclopropane-1-carboxylate (ACC) in *F.* × *ananassa* complete fruits (Upadhyay *et al.,* 2023), and in receptacles but not in achenes of *F. vesca* fruits (Gu *et al.,* 2019). This pattern is consistent with the expression of *ACC synthase* (*ACS*) and *ACC oxidase* (*ACO*) genes, which encode the enzymes involved in the last two steps of the ethylene biosynthesis pathway, respectively. Thus, the rise in ethylene content at early stages may be explained by the expression pattern of *FaACS1* and *FaACS2* in receptacles, and *FaACS3*, *FaACS4*, *FaACO2*, and *FaACO3* in achenes (Merchante *et al.,* 2013). The increase found in the ripening stages correlates with the up-regulation of *FaACO1* in *F.* × *ananassa* and *FveACO4b* in *F. vesca* receptacles (Merchante *et al.,* 2013; Gu *et al.,* 2019). Recently, Upadhyay *et al.* (2023) found 17 *ACS*-encoding genes in *F.* × *ananassa* with a ripening-related expression pattern, suggesting an important role for these genes in the regulation of the ethylene content during ripening.

The application of ethylene to strawberry fruits has not resulted in clear and consistent effects on ripening. However, it has been widely reported that ethylene treatment affects the expression of cell wall-related genes, such as those encoding β-galactosidase, pectin methylesterase, pectate lyase, β-xylosidase, and endoglucanase (Trainotti *et al.,* 2001; Castillejo *et al.,* 2004; Bustamante *et al.,* 2009; Villarreal *et al.,* 2016). The resulting changes in cell wall composition increase susceptibility to pathogens such as *Botrytis cinerea* and *Rhizopus stolonifera* (Villarreal *et al.,* 2016). A recent report has shown that ethylene treatment of *F. chiloensis* fruits at the large green developmental stage inhibited anthocyanin biosynthesis, but increased lignin content, which was consistent with the down-regulation of anthocyanin-related genes (*FcANS* and *FcUFGT*) and the up-regulation of *FcPOD27*, involved in lignin biosynthesis (Figueroa *et al.,* 2021a). In contrast, VIGS-mediated silencing of the product of the *S*-adenosyl-l-methionine (SAM) synthase-encoding gene *FaSAMS1*, which catalyzes the first committed step of the ethylene biosynthetic pathway, and the ethylene signaling gene *FaCTR1* in *F*. × *ananassa* receptacles resulted in an inhibition of fruit coloring, which was partially rescued by the synthetic ethylene ethephon (Sun *et al.,* 2013). Additionally, Merchante *et al.* (2013) generated stable transgenic lines overexpressing the Arabidopsis dominant negative allele of the ethylene receptor ETR (*etr1-1*). The insensitivity to ethylene resulted in an opposite trend in the expression of anthocyanin-related genes. Thus, *FaPAL* and *FaCHS* were slightly induced in receptacles, but significantly down-regulated in achenes, resulting in lighter achenes. These studies suggest that ethylene regulates fruit softening during ripening. However, further investigations are required to clarify the opposing effects of ethylene on anthocyanin accumulation that have been found to date, as well as to confirm the possible tissue-specific role of ethylene in anthocyanin biosynthesis.

In grape berries, only weak variations in endogenous ethylene levels occur around véraison (Fig. 1B) (Coombe and Hale, 1973; Chervin *et al.,* 2004). Thus, a brief increase of endogenous ethylene has been detected at the onset of grape ripening, which coincided with higher activity and transcripts levels of *VvACO1* (Chervin *et al.,* 2004), and the signal transduction-related genes *Ethylene Response 2* (*VvETR2*) and *Constitutive Triple Response 1* (*VvCTR1*) (Sun *et al.,* 2010). When the ethylene receptors were blocked with 1-methylcyclopropene (1-MCP) around the ethylene peak, a delay in the increase of berry diameter, a higher acidity level, and a transient inhibition of anthocyanin accumulation in berry skins occurred (Chervin *et al.,* 2004; Sun *et al.,* 2010). Furthermore, if grapes were treated with low doses of ethylene gas for 24 h just before véraison, the fruit diameter increased. Even after 1 h of application, early responses to ethylene are detected as the up-regulation of cell wall-related genes and water exchange genes mainly in the berry skins, including *expansin* genes (*EXP1/2*), *polygalacturonase* (*PG*), *cellulose synthase* (*CS*), *xyloglucan endotransglucosylase* (*XTH*), and *aquaporin* genes (*AQUA1–AQUA4*) (Chervin *et al.,* 2008). In contrast, treatment of berries at pre-véraison with high concentrations of either ethephon or the ethylene biosynthesis inhibitor AVG (aminoethoxyvinylglycine) results in a delayed or a faster beginning of ripening, respectively (Böttcher *et al.,* 2013b), suggesting that ethylene dosage affects whether it has a positive or negative role in the regulation of ripening. In summary, all these studies confirm that ethylene does play a role in non-climacteric ripening, mainly by regulating the biosynthesis of phenolic compounds and the modification of cell wall structure, and therefore the fruit softening process.

## ABA metabolism: ‘the dancing queen headlines the show’

**Fig. 2.**
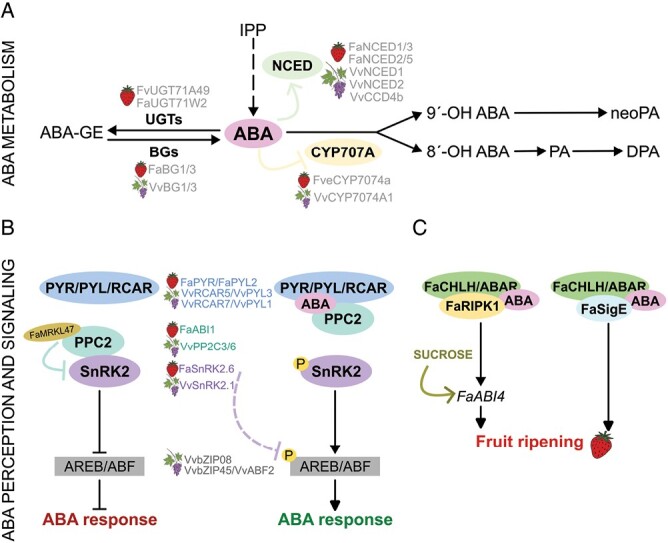
ABA metabolism and signaling in strawberry and grape berry fruits. (A) Schematic representation of ABA biosynthesis and catabolism pathways (oxidation and conjugation). Gray names represent enzymes involved in these processes. (B) Model of ABA perception and signaling in the absence or presence of ABA. (C) Novel signaling pathway described in strawberry. IPP, isopentenyl pyrophosphate; ABA, abscisic acid; UGTs, ABA-glucosyltransferases; BGs, β-glucosidases; ABA-GE, ABA-glucose ester; NCED, 9-*cis*-epoxycarotenoid dioxygenase; CYP707A, cytochrome P450 monooxygenase; PA, phaseic acid; DPA, dihydrophaseic acid; neoPA, neophaseic acid; PYR/PYL/RCAR, Pyrabactin Resistance 1 (PYR), PYR-like proteins (PYL), Regulatory Components of the ABA Receptors (RCAR); PPC2, type 2C protein phosphatases; FaMRKL47, FERONIA/FER-like receptor kinase (strawberry); SnRK2, Sucrose nonfermenting 1-related protein kinase 2; AREB/ABF, ABA-responsive element-binding protein; FaCHLH/ABAR, ABA receptor magnesium chelatase H subunit; FaRIPK1, Red-Initial Protein Kinase 1 (strawberry); FaABI4, ABSCISIC ACID-INSENSITIVE 4 (strawberry); FaSigE, Sigma factor E (strawberry).

ABA is commonly considered the key hormone controlling fruit ripening in non-climacteric fruits. However, it has a negative effect on the initial developmental stages (Liao *et al.,* 2018). The ABA content in fruits is controlled through the balance between biosynthesis and catabolism (Bai *et al.,* 2021; Figueroa *et al.,* 2021b). The early steps of ABA biosynthesis take place in the plastid as part of the MEP and carotenoid pathway. The first committed step in ABA biosynthesis is catalyzed by the 9-*cis*-epoxycarotenoid dioxygenase enzyme, encoded by *NCED*, which cleaves both 9ʹ-*cis*-violaxanthin and 9ʹ-*cis*-neoxanthin substrates to finally produce ABA (Fig. 2A). Two different ABA catabolism pathways have been described and are known to play a role in ripening regulation: (i) oxidative degradation and (ii) conjugation. The oxidative degradation of ABA is catalyzed by members of the cytochrome P450 monooxygenase (CYP707A) family. The predominant catabolic pathway involves the 8ʹ-hydroxylation of ABA, resulting in an unstable intermediate, 8ʹ-hydroxy-ABA. This is isomerized to phaseic acid (PA), which in turn is reduced to form dihydrophaseic acid (DPA) (Fig. 2A) (Rattanakon *et al.,* 2016; Figueroa *et al.,* 2021b). Oxidation of C-9ʹ can also occur, which is isomerized to neophaseic acid (neoPA) (Zhou *et al.,* 2004). The second catabolic pathway is the ABA conjugation. This pathway is controlled by ABA-glucosyltransferase (UGTs), producing the main conjugate of ABA, the ABA-glucose ester (ABA-GE). ABA-GE is considered an inactive product and the form in which ABA is transported and stored *in planta* (Figueroa *et al.,* 2021b). This pathway is reversible, as ABA-GE can be hydrolyzed to produce free and active ABA molecules through β-glucosidases (BGs) (Fig. 2A).

Several studies have shown that ABA content increases continuously throughout the ripening process in strawberries (Fig. 1A) (Chen *et al.,* 2011; Symons *et al.,* 2012; Gu *et al.,* 2019; Figueroa *et al.,* 2021b; Li *et al.,* 2022; Upadhyay *et al.,* 2023). Consistently, the levels of ABA catabolites 9ʹ-hydroxy-ABA and PA decrease during ripening (Upadhyay *et al.,* 2023). In contrast, no significant changes have been found for 7ʹ-hydroxy-ABA and neoPA during ripening (Figueroa *et al.,* 2021b).

ABA biosynthesis has been described as taking place mainly in the receptacles of *F. vesca*, being much less present in achenes (Gu *et al.,* 2019; Figueroa *et al.,* 2021b). However, a recent work has shown that both achenes and receptacles have similar ABA levels in *F.* × *ananassa* fruits (Li *et al.,* 2022). Nevertheless, this difference might be explained by the use of fresh instead of dry weight in Li’s work. In any case, ABA content is consistently low at the green stages and continuously increases during strawberry development and ripening.

Three or four members of *NCED* and three or five *CYP707A* genes are encoded in both *F. vesca* and *F.* × *ananassa* genomes, depending on the study (Kang *et al.,* 2013; Liao *et al.,* 2018; Li *et al.,* 2022). Furthermore, different nomenclatures for *NCED* genes has been followed, contributing to confusion. In 2011, Jia and collaborators showed that silencing the expression of *FaNCED1* [FvH4_3g16730; named *FveNCED3* in *F. vesca* (Liao *et al.,* 2018)] by VIGS in *F.* × *ananassa* fruits resulted in a pronounced reduction in the ABA content and delayed fruit ripening (Jia *et al.,* 2011). This phenotype was rescued when the fruits were treated with exogenous ABA, providing evidence for the first time of the promoting role of ABA in the ripening process. However, shortly after, it was shown that the expression of *FaNCED2* [FvH4_3g05440; named *FveNCED5* in *F. vesca* (Liao *et al.,* 2018)] was much higher than that of *FaNCED1* (Ji *et al.,* 2012). This higher expression was confirmed in further transcriptome studies (Sánchez-Sevilla *et al.,* 2017; Martín-Pizarro *et al.,* 2021), suggesting a major role for this gene in ABA biosynthesis in strawberry fruits.

ABA biosynthesis in strawberry receptacles is known to be a tightly controlled process in which a crosstalk between ABA, auxin, and GAs in achenes and receptacles is essential. Expression data showed that, at early fruit developmental stages, auxin and GA induce the expression of *FveCYP707A4a* in receptacles, ensuring that the endogenous ABA content is kept low during the growth phase (Liao *et al.,* 2018). Once the fruit has reached the precise size, auxin and GA content decline and the expression of *FveCYP707A4a* is reduced, releasing the activation of ABA biosynthesis, which in turn inhibits IAA biosynthesis (Liao *et al.,* 2018; Li *et al.,* 2022). Thus, when *FveCYP707A4a* was transiently silenced at early stages, the expression of *NCED* genes was found to be up-regulated, and ABA levels increased in immature fruits (Liao *et al.,* 2018). These fruits displayed lower fruit firmness than the control, which is explained by the high expression of genes responsible for cell wall disassembly, such as *pectate lyase* (*FvePL*) and *endo-β-1,4-glucanase* (*FveCEL2*). Furthermore, ABA treatment repressed *FveCYP707A4a* and promoted *NCED* gene expression, resulting in a regulatory feedforward loop to control ABA content in receptacles (Liao *et al.,* 2018).

It has been recently suggested that the decline in auxin biosynthesis in achenes at the onset of ripening might be due to an autocatalytic ABA biosynthesis in this tissue. The application of ABA to detached achenes and receptacles increased ABA levels and reduced that of IAA in both tissues; however, in achenes, ABA biosynthesis was significantly promoted, supporting an ABA regulatory feedforward loop in the latter (Li *et al.,* 2022). This study also suggested a more important role for ABA accumulation in achenes than in receptacles for strawberry fruit ripening regulation. Li *et al.* (2022) found ripening induction after ABA treatments via stalk feeding, rather than receptacle injection. Since ABA seems to be accumulated mainly in achenes or in receptacles with each method, respectively, they concluded that ABA in achenes plays a major role in ripening regulation. However, ABA treatment via injection in receptacles has been widely used with positive results (Table 1) (Jia *et al.,* 2011; Li *et al.,* 2019; Luo *et al.,* 2020; Martín-Pizarro *et al.,* 2021). Therefore, although the role of ABA in achenes seems to be important, the reasons for this different effect on ripening via ABA infiltration are not clear and might be explained by methodological differences.

Members of the ABA conjugation catabolic pathway regulating strawberry ripening have also been studied. In particular, two BGs have been characterized, *FaBG3* (Li *et al.,* 2013) and *FaBG1* (Zhang *et al.,* 2014). When their expression was silenced by RNAi, a reduction in the ABA content and an inhibition in the ripening process occurred, demonstrating the importance of ABA-GE hydrolysis, to release active ABA in the receptacle. However, differences in the levels of ABA-GE conjugate were found when comparing *F. vesca* accessions and one *F.* × *ananassa* cultivar. ABA-GE content decreased only in the octoploid accession, and increased in all the diploid accessions, suggesting differences in ABA catabolism that might be caused by the polyploid nature of *F.* × *ananassa* (Figueroa *et al.,* 2021b). Furthermore, they found that FvUGT71A49 was able to glycosylate ABA more efficiently than other UGTs and that the overexpression of this gene in fruits produced a reduction in free ABA but not in ABA-GE content in receptacles. Similarly, ABA-GE levels did not change significantly when another *UGT* gene, *FaUGT71W2*, was down-regulated, which might be explained by compensation due to the redundancy of the ABA UGTs. Taken together, all these results are evidence that the ripening process in strawberries is controlled by endogenous ABA content, which at the same time is controlled by a tight balance between biosynthesis and catabolism.

ABA also plays a key role in the regulation of grape berry ripening (Pilati *et al.,* 2017). As in strawberry, the ABA level rises before véraison, during ripening, but in contrast to strawberry, it declines during the last ripening stages (Fig. 1B) (Zhang *et al.,* 2009; Zhang *et al.,* 2013). Exogenous ABA application to different grape cultivars at véraison hastens the ripe state. This is the result of an increase in the expression of genes involved in the synthesis of anthocyanins, which color the skin berry rapidly after the treatment, as well as in the total phenolic content, and the concentration of flavonoids and antioxidants, while organic acids decrease and berry weight and volume do not change (Ban *et al.,* 2003; Jeong *et al.,* 2004; Peppi *et al.,* 2006, 2007; Cantín *et al.,* 2007; Giribaldi *et al.,* 2010; Koyama *et al.,* 2010; Wang *et al.,* 2016). Nevertheless, treatment with exogenous ABA before véraison (at the hard green stage) results in long-term effects, since the ABA content of the treated berries remains higher not only immediately after its application, but also nearly 40 d later (Villalobos-González *et al.,* 2016), modulating significantly several genes involved in ABA biosynthesis, perception, and signaling (Wheeler *et al.,* 2009; Pilati *et al.,* 2017).

As in strawberries, ABA content is controlled by the rate of biosynthesis and catabolism of this hormone in grape fruits. Three *NCED* isoforms in grapevine have been found (Young *et al.,* 2012), which differ in their expression pattern and, as in strawberry, have been named differently depending on the study. However, there is a consensus that, in most grape cultivars, the *VvNCED1* isoform is associated with the beginning of ripening. The expression levels of *VvNCED1* (VIT_219s0093g00550; named *VvNCED1* in Wheeler *et al.,* 2009; Zhang *et al.,* 2009; Sun *et al.,* 2010; Zhang *et al.,* 2013; Pilati *et al.,* 2017; and *VvNCED3* in Young *et al.,* 2012; Wong *et al.,* 2016) were low at the early developmental stages but increased at the onset of ripening, mainly in the seeds and skin, but also in the pulp, and declined in later ripening stages, except in the skin (Zhang *et al.,* 2013), which is consistent with the timing and pattern of ABA accumulation. In contrast, *VvNCED2* expression seems to be up-regulated at the later stages of véraison (Lund *et al.,* 2008; Young *et al.,* 2012), and also 44 h post-ABA treatment (Pilati *et al.,* 2017), as well as expression of *VvNCED4*, which is also modulated during post-harvest withering (Wong *et al.,* 2016; Pilati *et al.,* 2017). Finally, the *NCED* isoform *VvCCD4b* appears to be induced during the whole berry ripening in all tissues, although slightly more in the pulp (Pilati *et al.,* 2017).

The expression levels of the ABA degradation enzyme gene *VvCYP707A1* negatively correlate with *VvNCED1* transcripts and ABA content, suggesting that it might work as a negative regulator and a possible fine-tuner of the ABA content (Zhang *et al.,* 2013). The conjugate ABA-GE levels also decline during ripening (Owen *et al.,* 2009), consistent with the expression of *VvGT*, which peaks around véraison like *VvNCED1*, although its expression level was lower (Sun *et al.,* 2010). Finally, the expression of *VvBG* genes showed different patterns in the different developmental and ripening stages (Zhang *et al.,* 2013). Transcript levels of *VvBG1* and *VvBG2* were low at the early developmental stages, and increased from coloration to fruit ripening, coinciding with ABA accumulation, suggesting a role for these two enzymes in controlling ABA content (Sun *et al.,* 2010; Zhang *et al.,* 2013). Furthermore, VvBG1 seems to have a more important role than VvNCED1 in ABA content regulation at late developmental stages, since *VvBG1* expression remained at high levels longer than that of *VvNCED1* (Sun *et al.,* 2010). In contrast, transcript levels of *VvBG3* were high during early grape growth and decreased gradually throughout development, suggesting that it might influence ABA content in the early stages (Sun *et al.,* 2010; Zhang *et al.,* 2013).

## ABA signaling: ‘the dancing queen gets the crowd to sing along’

The ABA signal transduction pathway is essential for this phytohormone to play its regulatory role. Two different signaling pathways exist, the first one constituted by three major components: PYR/PYL/RCAR (ABA receptors), clade A protein phosphatase 2C (PP2Cs, negative regulators), and sucrose-nonfermenting kinase 1-related protein kinase 2 (SnRK2s, positive regulators) (Fig. 2B). In the presence of ABA, the PYR/PYL/RCAR cytosolic receptors change their conformation and bind to PP2C, inhibiting its activity and releasing SnRK2s. In turn, SnRK2 is phosphorylated, activating TFs of the ABA-responsive element-binding factor subgroup (AREB/ABF) to mediate transcription of ABA-responsive genes (Fig. 2B) (Wang *et al.,* 2013). Various studies have been performed to understand the molecular mechanism underlying ABA perception and signaling (Chen *et al.,* 2020). In strawberries, nine members of both *FaPYR/PYL* and *FaPP2C* gene families have been identified (Hou *et al.,* 2021). Transient up- and down-regulation of different members of these families have been performed to study their function. Firstly, down-regulation of the ABA receptor *FaPYR* in *F.* × *ananassa* promoted a loss in receptacle color, which could not be restored by exogenous ABA treatment (Chai *et al.,* 2011). In contrast, silencing of the PP2C1-encoding gene *ABSCISIC ACID INSENSITVE 1* (*FaABI1*) promoted fruit ripening, while its overexpression had the opposite effect (Jia *et al.,* 2013b). These data support a positive and a negative role for *FaPYR* and *FaABI1*, respectively, in the regulation of strawberry fruit ripening. It has been recently found that FaABI1 also interacts with FaPYL2, which might be relevant in the control of ripening due to the ripening-induced expression pattern of *FaPYL2* (Hou *et al.,* 2021). Furthermore, FaABI1 interacts with the FERONIA/FER-like receptor kinase FaMRKL47. Overexpression and RNAi-mediated silencing of *FaMRKL47* delayed and accelerated strawberry fruit ripening, respectively, supporting a negative role for FaMRKL47 in ABA signaling (Jia *et al.,* 2017). Another component of this pathway is FaSnRK2.6. RNAi silencing of *FaSNRK2.6* significantly promoted ripening, while its overexpression arrested it, supporting a negative role in the regulation of this process (Han *et al.,* 2015). A direct interaction was also found between FaSnRK2.6 and FaABI1, although it is hypothesized that the mechanism by which FaSnRK2.6 regulates ABA-mediated strawberry fruit ripening is at the transcriptional and not at the post-transcriptional level, since the ABA/FaPYR1/FaBI1 signaling cascade would be expected to activate rather than deactivate FaSnRK2.6. This hypothesis is supported by the decrease in the transcript levels of *FaSNRK2.6* that has been observed during development and ripening, and after ABA treatment (Han *et al.,* 2015).

A second pathway has been described in strawberry (Fig. 2C). A putative ABA receptor gene, *FaCHLH/ABAR* (also named *FaABAR* in Hou *et al.,* 2018), was identified. Its down-regulation by RNAi resulted in an uncolored fruit phenotype, despite containing a higher ABA content than control fruits. Moreover, exogenous ABA application could not restore the fruit color, supporting a blockage of the ABA signaling pathway and a feedback effect on the ABA content when this pathway is repressed (Jia *et al.,* 2011). These fruits exhibited lower sugar content, probably due to the up-regulation of *Sigma factor E* (*FaSigE*), a regulator of sugar catabolism, and *α-amylase* (*FaAMY*) (Jia *et al.,* 2011). Consistently, the RNAi silencing of *FaSigE* in *F.* × *ananassa* produced firmer fruits than the controls, with a reduction in soluble solids, anthocyanins, and ABA content, indicating that *FaSigE* is a positive regulator of fruit ripening (Zhang *et al.,* 2017). Interestingly, *FaABAR* expression and ABA content were down-regulated in the *FaSigE* RNAi fruits, and the interaction between FaABAR and FaSigE was confirmed. All these data suggested that *FaSigE*, *FaABAR*, sugars, and ABA are involved in the pathway controlling fruit ripening (Zhang *et al.,* 2017). Another member of this pathway has been found with the identification of the protein interaction between FaABAR and Red-Initial Protein Kinase 1 (FaRIPK1) (Hou *et al.,* 2018). *FaRIPK1* function was analyzed by transient down-regulation in *F*. × *ananassa*, which resulted in an inhibition of strawberry fruit ripening and a reduced expression of genes involved in softening, sugar content, anthocyanin biosynthesis, and ABA biosynthesis and signaling, including *FaNCED1* and the TF-encoding *FaABI4* gene. Thus, in this model, ABA binds to the complex constituted by FaABAR and FaRIPK1, promoting fruit ripening through the modulation of *FaABI4* gene expression (Hou *et al.,* 2018). Whether FaSigE and FaRIPK1 are part of the same protein complex with FaABAR is yet to be elucidated.

In grapevine, nine ABA receptors of the VvPYL/PYR/RCAR family, 85 VvPP2Cs, and eight SnRK2 kinases have been described, with organ-specific expression patterns (Boneh *et al.,* 2012a, b; Liu *et al.,* 2016; Zhang *et al.,* 2021). An interesting correlation between gene expression and the interaction of ABA receptors with PP2Cs has been described (Gambetta *et al.,* 2010; Boneh *et al.,* 2012b; Pilati *et al.,* 2017). In berry fruits, exogenously applied ABA at the hard green pre-véraison stages down-regulated the expression of *VvRCAR5* (VIT_208s0058g00470) and *VvRCAR7* (VIT_202s0012g01270) (named *VlPYL3* and *VlPYL1*, respectively, in Li *et al.,* 2011) (Pilati *et al.,* 2017). *VvRCAR7/VlPYL1* is mainly expressed in leaves and fruit tissues. It increases during fruit development and declines before ripening, correlating with ABA levels (Gao *et al.,* 2018). Furthermore, transient overexpression of *VvRCAR7/VlPYL1* in grape berries enhanced color development and anthocyanin accumulation in the skin, supporting a positive role in this biosynthetic pathway (Gao *et al.,* 2018). The expression pattern of the negative regulators, the *VvPP2C* genes, showed that *VvPP2C3* and *VvPP2C6* were up-regulated during the ripening stages, suggesting a role for these two genes in ABA signaling during grape berry ripening (Gambetta *et al.,* 2010; Pilati *et al.,* 2017). Moreover, the protein kinase VvSnRK2.1 is considered a candidate to be part of this ABA signaling pathway in grape berries, since its transcript levels increase after ABA exogenous application (Pilati *et al.,* 2017). Among the downstream AREB/ABFs TFs, only two VvABFs have been identified in grapevine: VvbZIP08 and VvbZIP45/ABF2 (Liu *et al.,* 2014). In particular, the transcript levels of *VvAREB/ZIP45/ABF2* increased from véraison until the end of the ripening phase, mainly in both seeds and skin, and after exogenous ABA treatment (Nicolas *et al.,* 2014; Wong *et al.,* 2016; Pilati *et al.,* 2017). Moreover, the overexpression of *VvAREB/ZIP45/ABF2* strongly increased the accumulation of stilbenes and activated processes related to fruit softening and ripening, indicating that this TF is the main phosphorylated intermediary between SnRK2 and downstream genes of the ABA signaling cascade such as *VvNAC17* and *Armadillo-like*, expression of which was also induced by ABA treatment and by VvAREB/ZIP45/ABF2 in trans-activation assays in tobacco leaves (Nicolas *et al.,* 2014; Pilati *et al.,* 2017). Finally, VviNAC060 has been identified as a positive regulator of grape ripening, as it regulates the ABA transduction pathway and promotes chlorophyll degradation and anthocyanin accumulation (D’Incà *et al.* 2023).

A crosstalk between ABA and auxins, GAs, and ethylene has been described in grapevine, where auxin and GA_3_ application caused a decrease in the cellular ABA levels by inhibiting the synthesis via down-regulation of *VvNCED* genes (Zilioto *et al.,* 2012; Chai *et al.,* 2014). Furthermore, auxin and GA_3_ application also modulated ABA signaling by decreasing the transcript levels of the *VvPP2C* genes and the TF-encoding *VvABF* gene (Zilioto *et al.,* 2012; Chai *et al.,* 2014). In contrast, ethylene and ABA are part of a feedforward loop promoting the expression of *VvNCED1* and *VvACO1*, respectively (Sun *et al.,* 2010).

The positive role of ABA has also been described in the regulation of ripening of other non-climacteric fruits, such as lychee, in which ABA treatment promotes anthocyanin accumulation mediated by several TFs such as LcABFs, LcMYB1, LcbHLH1, and LcNAC002 (Singh *et al.,* 2014; Hu *et al.,* 2019; Zou *et al.,* 2023). The same role has also been identified in sweet cherry, in which ABA signaling is regulated by DOF TFs, such as PavDof2, and PavDof6/15, with opposite roles in the regulation of cell wall-related and ABA biosynthetic genes (Zhai *et al.,* 2022), and PaMADS7 or PavNAC56, which promote fruit softening (Qi *et al.,* 2020, 2022). The importance of ABA regulating fruit ripening and ABA-related TFs has also been reported in *Citrus reticulata* and *Citrus sinensis* (Zhu *et al.,* 2020). In summary, all these data together confirm that, despite the different fruit types, ABA is a common and important regulator in the promotion of ripening in non-climacteric fruits.

## Conclusions

We have presented a comprehensive review on the hormonal regulation of non-climacteric fruit ripening, focusing on the two main model species, strawberry and grapevine. We have highlighted the importance of auxin and GAs in the early stages of fruit development and their role in inhibiting ripening. Furthermore, the data discussed here are evidence that ABA, our ‘dancing queen’, is the main hormone positively regulating non-climacteric fruit ripening. Recently, an interesting autocatalytic biosynthesis of ABA has been described in strawberry, in which achenes seem to play a much greater role than has been previously considered (Li *et al.,* 2022). However, the differences found in some reports concerning ABA levels in achenes and receptacles, as well as in the ripening effect after ABA infiltration, need to be reconsidered before drawing further conclusions. In any case, the major role of ABA is undeniable. However, ABA should not ‘take all the credit for the show’ since other hormones such as ethylene and BRs also contribute to reaching the final ripe stage in non-climacteric fruits.

Here we have discussed the similarities and differences between the hormone accumulation pattern in strawberries and grapes. We have also described the effects of these hormones on fruit development and ripening, as well as the main genes involved in both biosynthesis and signaling. All these data highlight the different nature of strawberries and grape berries. Strawberry has become the main model for non-climacteric fruits, despite it developing false fruits derived from vegetative tissue in contrast to the ovary-derived grape berry. It is important to take this into account, and results obtained in strawberry fruit receptacles should not be directly extrapolated to other non-climacteric species with ovary-derived fruits. Furthermore, ethylene increases and a respiratory burst even occurs in the late stages of strawberry ripening (Iannetta *et al.,* 2006), which should invite the scientific community to reconsider the classification of fruit ripening into more categories than just climacteric and non-climacteric.

Although not the focus of this review, it is worth mentioning that the role of TFs and environmental factors in the regulation of fruit development and ripening is essential. Several key TFs that are regulated by ABA and/or regulate ABA biosynthesis and signaling have been identified so far in non-climacteric fruits, such as the NAC TFs FaRIF and VviNAC060 (Martín-Pizarro *et al.,* 2021; D’Incà *et al.,* 2023), or FaGAMYB and VvAREB/ZIP45/ABF2 (Nicolas *et al.,* 2014; Vallarino *et al.,* 2015) in strawberry and grapevine, respectively, among others. Therefore, there is a chicken-and-egg situation here: what is the most upstream molecular signal to trigger fruit ripening? Is it actually an epigenetic modification instead? This open question is still unresolved and will be complicated to answer.

Besides the key role of ABA in promoting non-climacteric fruit ripening, this phytohormone is essential to deal with abiotic stresses. Therefore, it is not unexpected that salt and drought stresses induce the expression of ABA biosynthetic genes, and therefore a rise in ABA accumulation and, subsequently, in the anthocyanin content in *F*. × *ananassa* fruits (Galli *et al.,* 2016; Perin *et al.,* 2019). In grapes, water deficit alters the volatile composition of the berries (Buesa *et al.,* 2021), and also promotes ripening, increasing the anthocyanin levels as the result of an up-regulation of genes of the anthocyanin biosynthetic pathway (Castellarin *et al.,* 2007). Temperature is another environmental factor influencing fruit ripening. In strawberry, high temperature dramatically accelerates fruit ripening, which seems to be mediated by the ABA negative regulator FaSnRK2.6 (Han *et al.,* 2015). In contrast, high temperatures negatively affect the maturation process in grapes by accelerating anthocyanin degradation and inhibiting anthocyanin biosynthesis in berry skin (Mori *et al.,* 2005a, b, 2007), which is consistent with the reduction found in ABA levels at high temperatures (Ryu *et al.,* 2020). Thus, it is clearly important to study further the molecular mechanisms underlying the response to the abiotic stresses associated with the current context of climate change, providing gene targets which when modulated will allow optimization of fruit set and ripening and improve fruit quality traits. However, this requires the development of efficient transformation protocols.

Strawberry has a great advantage over other non-climacteric species, since it allows gene functional analysis to be performed in a rather simple way by either transient transformation of the receptacles or stable plant transformation. Unfortunately, this is not easy to achieve, or is more time-consuming, in other non-climacteric species, as in the case of grapevine, and tree species such as citrus, which are recalcitrant for generation of stable transgenic plants. Nevertheless, stable transformation has been achieved in grapevine (Rinaldo *et al.,* 2015; D’Incà *et al.,* 2023), and new strategies are being developed in order to optimize the regeneration problems in this species (Campos *et al.,* 2021). Importantly, although it is not a straightforward methodology and requires proper controls, transient transformation by agroinfiltration can be directly performed in the strawberry receptacles and grape berries. This facilitates studying the role of genes of interest for fruit ripening *in situ* and is much faster than obtaining stable transgenic plants (Carvalho *et al.,* 2016; Gao *et al.,* 2018; Zhao *et al.,* 2019). In spite of the advantages of transient transformations, we believe that the development of efficient stable transformation methodologies will be essential to gain further and more accurate knowledge on the genetic mechanisms underlying non-climacteric fruit ripening, allowing optimization of the fruit quality traits acquired during this process, as well the resistance to pathogens, and thus obtaining more nutritious, flavorful, and longer lasting fruits in the near future for all these important crops.

## Data Availability

This review contains no new experimental data
